# Cloning and Expression Analysis of *Vvlcc3*, a Novel and Functional Laccase Gene Possibly Involved in Stipe Elongation

**DOI:** 10.3390/ijms161226111

**Published:** 2015-12-01

**Authors:** Yuanping Lu, Guangmei Wu, Lingdan Lian, Lixian Guo, Wei Wang, Zhiyun Yang, Juan Miao, Bingzhi Chen, Baogui Xie

**Affiliations:** 1 Mycological Research Center, College of Life Sciences, Fujian Agriculture and Forestry University, Fuzhou 350002, China; yuanplu1106@163.com (Y.L.); Letitiaddan@163.com (L.L.); LXG20150331@163.com (L.G.); uniwangwei@163.com (W.W.); zhiyunyang1@126.com (Z.Y.); 18396511363@163.com (J.M.); cbz_2006@163.com (B.C.); 2 College of Horticulture Sciences, Fujian Agriculture and Forestry University, Fuzhou 350002, China; wgm_2014@163.com

**Keywords:** *Volvariella volvacea*, laccase, fruiting body formation, enzyme

## Abstract

*Volvariella volvacea*, usually harvested in its egg stage, is one of the most popular mushrooms in Asia. The rapid transition from the egg stage to elongation stage, during which the stipe stretches to almost full length leads to the opening of the cap and rupture of the universal veil, and is considered to be one of the main factors that negatively impacts the yield and value of *V. volvacea*. Stipe elongation is a common phenomenon in mushrooms; however, the mechanisms, genes and regulation involved in stipe elongation are still poorly understood. In order to study the genes related to the stipe elongation, we analyzed the transcription of laccase genes in stipe tissue of *V. volvacea*, as some laccases have been suggested to be involved in stipe elongation in *Flammulina velutipes*. Based on transcription patterns, the expression of *Vvlcc3* was found to be the highest among the 11 laccase genes. Moreover, phylogenetic analysis showed that VvLCC3 has a high degree of identity with other basidiomycete laccases. Therefore, we selected and cloned a laccase gene, named *Vvlcc3*, a cDNA from *V. volvacea*, and expressed the cDNA in *Pichia pastoris*. The presence of the laccase signature L1-L4 on the deduced protein sequence indicates that the gene encodes a laccase. Phylogenetic analysis showed that VvLCC3 clusters with *Coprinopsis* cinerea laccases. The ability to catalyze ABTS (2,2’-Azino-bis(3-ethylbenzothiazoline-6-sulfonic acid) oxidation proved that the product of the *Vvlcc3* gene was a functional laccase. We also found that the expression of the *Vvlcc3* gene in *V. volvacea* increased during button stage to the elongation stage; it reached its peak in the elongation stage, and then decreased in the maturation stage, which was similar to the trend in the expression of *Fv-lac3* and *Fv-lac5* in *F. velutipes* stipe tissue. The similar trend in expression level of these laccase genes of *F. velutipes* suggested that this gene could be involved in stipe elongation in *V. volvacea*.

## 1. Introduction

*Volvariella volvacea* (Bull.: Fr. Sing.) is an economically important edible mushroom; it is a straw-degrading basidiomycete that has been cultivated extensively in the southern provinces of China for several centuries. Generally, *V. volvacea* is harvested in the egg stage [1] during which the fruiting body of *V. volvacea* is egg shaped with a pileus and stipe that are still hidden by the universal veil [2]. While the stipe rapidly extends to almost full length in the elongation stage, the pileus is fully opened in the mature stage [2]. *V. volvacea* harvested in its egg stage has a longer shelf life, better nutritious value and premium price compared to later developmental stages [3]. In other words, the quality of *V. volvacea* is influenced by the rupture of the universal veil and opening of the cap. However, very little is known about the mechanisms and regulation involved in stipe elongation. *V. volvacea*, completes its cropping cycle within three weeks [4], and is a good model to investigate the mechanism of stipe elongation due to the fast growth rate.

Previous studies have revealed some genes involved in stipe elongation, such as genes encoding chitin synthases [5], a septin protein and Cytochrome P450 [6,7], which are related to elongation of mushroom stipe. Recently, Wang *et al*., reported that some laccases are involved in stipe elongation in *Flammulina velutipes* [8]. Laccases (benzenediol: oxygen oxidoreductases, EC 1.10.3.2) belong to a family of blue multicopper oxidases that catalyze the oxidation of a variety of aromatic substrates along with the reduction of molecular oxygen to water [9]. The multigene family of laccases is a common feature in fungi [9]. The first example of the multigene family of laccases was described in *Agaricus bisporus* [10]. Since then, two laccases have been characterized from *Pycnoporus cinnabarinus* [11], 11 genes from *Trametes versicolor* [12], 17 from *Coprinopsis cinerea* [13], three from *Pleurotus eryngii* [14], 11 laccases from *Laccaria bicolor* [15], 12 from *Pleurotus ostreatus* [16] and 11 from *Flammulina velutipes* have been characterized [8]. Fungal laccases have been associated with formation of pigment during asexual development [17], fruiting body formation [18], and lignin degradation [19]. Laccases are multi-functional enzymes and most of their roles are not yet understood (see review by Kües *et al.* [20]). We hypothesized that some laccases could be involved in stipe elongation in *V. volvacea* as has been suggested for *F. velutipes*.

In this study, we analyzed the expression of *V. volvacea* laccase genes in different stages of sporophore development. We then cloned the *Vvlcc3* gene from *V. volvacea*, the strongest expressed laccase gene during fruiting, and successfully expressed the *Vvlcc3* cDNA in *Pichia pastoris.* The expression pattern of this gene was similar to that of *Fv-lac3* and *Fv-lac5* in the stipe of *F. velutipes* during fruiting body development, suggesting that *Vvlcc3* could be important for stipe elongation in *V. volvacea*.

## 2. Results

### 2.1. Laccase Genes in the V. volvacea Genome and Their Transcription Patterns

**Figure 1:**
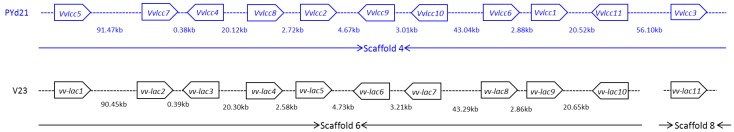
Distribution of laccase genes in the genome of *V. volvacea* strain PYd21 and V23. Arrow-shaped boxes indicate the laccase genes, whereas the direction of arrows shows the direction for each gene. The numerical values below the line indicate the distances (bp) between pairs of laccase genes. Blue boxes indicate laccase genes from the PYd21; black boxes indicate laccase genes from V23.

Based on the homology search results using local BLAST analysis by comparing the sequence of six laccase genes from *V. volvacea* V14 downloaded from NCBI (national center for biotechnology information) to the genome of *V. volvacea* PYd21 (GenBank no: ANCH00000000.1), 11 putative laccase genes were found in the PYd21 genome. Among them, six laccase amino acid sequences were more closely matched with the previously cloned laccase genes in *V.volvacea*, namely, *Vvlcc1* (AY249052.1) [21], *Vvlcc2* (AY338483.1) [18], *Vvlcc3* (AY338484.1), *Vvlcc4* (AY338486.1), *Vvlcc5* (AY338485.1), and *Vvlcc6* (AY338487.1). The other five laccase genes named *Vvlcc7-* *Vvlcc11* followed the order on the scaffolds (PYd21 in Figure 1). In addition, Bao *et al*., [22] published the multigene family of laccases in the *V. volvacea* strain V23. They also named the laccase genes in V23 using the order on the scaffolds. We aligned the laccase gene loci between PYd21 and V23; 11 laccase genes exhibited similar loci in both genomes (Figure 1), which indicated that the loci structure in *V. volvacea* is conserved. Furthermore, all of these genes from the PYd21 genome exhibited high homology with the sequences retrieved from *V. volvacea* V23 (Table S1. Identity ≥ 98%, *E*-value = 0).

For gene expression analysis, tags (Table S2) were mapped solely to cDNA of every laccase gene for each developmental stage and normalized to Transcripts per Million clean tags (TPM) [23]. Differences in the expression levels of these genes were detected when compared to different developmental stages (Table 1). All of the laccase genes, except *Vvlcc7*, *Vvlcc8* and *Vvlcc10*, were expressed in at least one stage. Among these genes, *Vvlcc3* exhibited the highest expression levels in every sample, and it also showed an interesting expression pattern, thus *Vvlcc3* was chosen for further study.

**Table 1:** Differential expression levels of the laccase genes at different developmental stages (from button stage to maturation stage). BU (Button stage): the stage after inoculating spawn 10 days on rice straw compost; EG (egg stage): the stage after inoculating spawn 13 days; EL (elongation stage): the stage after inoculating spawn 13.5 days; MA (maturation stage): the stage after inoculating spawn 14 days.

	*Vvlcc* *1*	*Vvlcc* *2*	*Vvlcc* *3*	*Vvlcc* *4*	*Vvlcc* *5*	*Vvlcc* *6*	*Vvlcc* *7*	*Vvlcc* *8*	*Vvlcc* *9*	*Vvlcc* *10*	*Vvlcc* *11*
BU	0	1.96	8.73	4.99	0.36	0	0	0	0.18	0	0.89
EG	0.33	0.33	71.09	14.55	3.34	0.50	0	0	1.50	0	1.34
EL	0	0	92.20	9.88	0	0.67	0	0	1.17	0	0.50
MA	0	1.17	27.14	11.23	0	1.01	0	0	0.50	0	0

**Figure 2:**
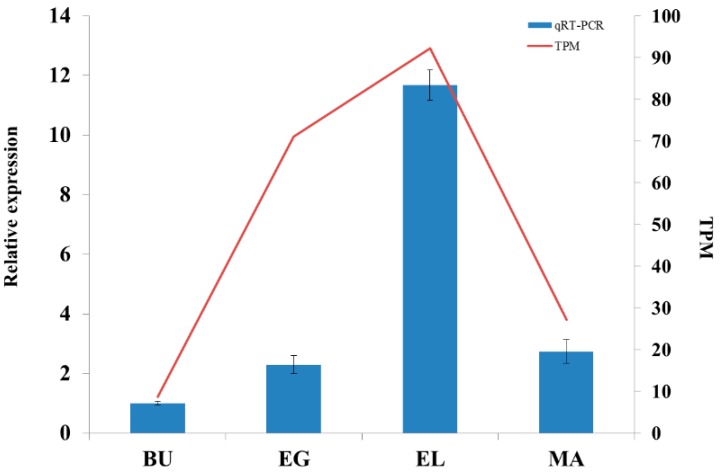
The expression analysis of *Vvlcc3* using DGE and qRT-PCR. TPM: Transcripts per Million clean tags. BU (Button stage): the stage after inoculating spawn 10 days on rice straw compost; EG (egg stage): the stage after inoculating spawn 13 days; EL (elongation stage): the stage after inoculating spawn 13.5 days; MA (maturation stage): the stage after inoculating spawn 14 days.

Low transcript levels of *Vvlcc3* analyzed based on the digital gene expression (DGE) data were detected in the button stage. The expression of *Vvlcc3* then increased at the egg stage and peaked at the elongation stage. The expression level of *Vvlcc3* decreased at the mature stage. These results were further confirmed by qRT-PCR (Figure 2). The transcription pattern of *Vvlcc3* strongly suggests that the function of this gene may be related with the elongation stage.

### 2.2. The Structure of Vvlcc3 Gene

**Figure 3:**
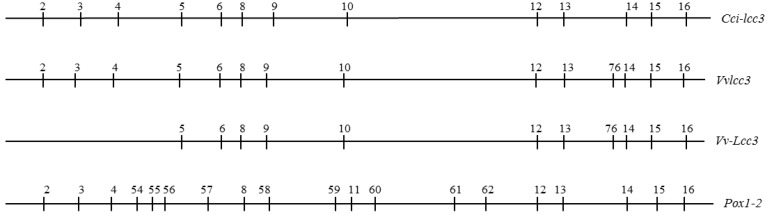
Relative position of introns in different laccase genes; nomenclature of introns referred to Ahlawat 2011 [25]. *Cci-lcc3*: *C.cinerea lcc3* (AF118269.1); *Vvlcc3*: *V. volvacea* strain PYd21 *lcc3* (*KF365491.1*); *Vv-lcc3*: *V. volvacea* strain VV1 *lcc3* (HQ687205); *Pox1-2*: *P. ostreatus Pox1* (Q12729.1) and *Pox2* (Q12739.1)

Based on *V. volvacea* whole genome sequencing data from strain PYd21, primers (lcc3OF and lcc3OR) were designed to clone *Vvlcc3* cDNA. The full-length cDNA of *Vvlcc3* consisted of 1548 bp. Alignment of the *Vvlcc3* genomic DNA sequence and cDNA sequence using DNAMAN (version 5.2.2) revealed that the coding region was interrupted by 14 introns (Figure 3). The size of the 14 introns ranged from 50 to 80 bp, and all of the splice junctions of the introns conformed to the GT-AG rule [24]. Comparison of the structure of *Vvlcc3* with *vv-lac11* as reported by Bao *et al.* (2013) indicates the same number of introns [22]. Comparison of the structure of *Vvlcc3* with *Vv-Lcc3* reported by Ahlawat in 2011 [25] confirmed that the first three introns were found. The intron positions were determined and compared with intron positions in *Coprinopsis cinerea Cci-lcc3* and *Pleurotus ostreatus Pox1-2* (Figure 3), indicating a high similarity between *Vvlcc3* and *Cci-lcc3*, since only one position (76) is absent in the *Cci-lcc3* gene. Moreover, *Vvlcc3* and *Cc-lcc3* have similar intron positions and share position 5, 9 and 10 introns, which are absent in *P. ostreatus Pox1* and *Pox2*.

### 2.3. Characterization of VvLCC3 Protein and Phylogenetic Tree

**Figure 4:**
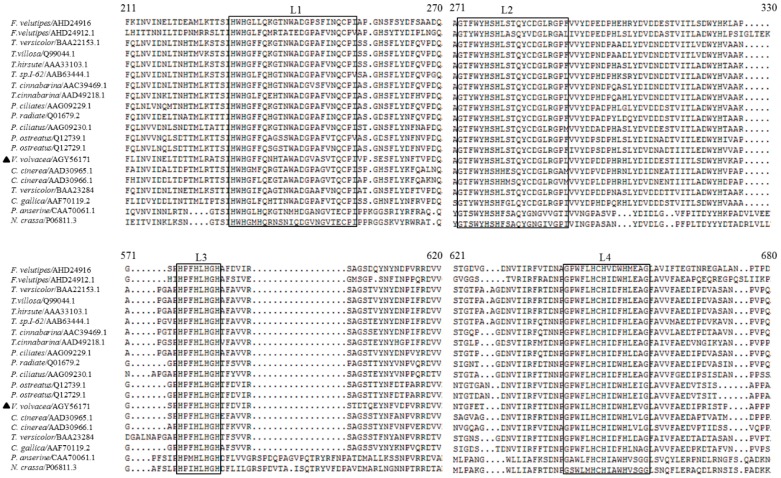
Alignment of the deduced amino acid sequence of VvLCC3 with homologous laccases using Clustal X (1.8). Four black boxes indicate four fungal laccase signature sequences (L1-L4), including 10 conserved histidine residues and one cysteine residue. The triangle indicates *V. volvacea* VvLCC3 protein.

The product of *Vvlcc3* consisted of 515 amino acids containing a mature protein of 496 amino acids and a putative signal peptide of 19 amino acids. We found that the theoretical isoelectric point (pI) of VvLCC3 was 4.62 using ExPASy Protparam. An InterProScan search indicated that the deduced protein contained three multicopper oxidase domains (type 1, IPR001117; type 2, IPR011706; type 3, IPR011707). Three *N*-glycosylation sites (Asn-Xaa-Thr/Ser, in which Xaa is not Pro), at positions 89, 114, and 451 of the deduced amino acids, were found using NetNGlyc 1.0 Server, suggesting that VvLCC3 from *V. volvacea* is a glycoprotein. The alignment of the deduced amino acid sequence indicated four copper-binding sites (L1–L4), including 10 conserved histidine residues and one cysteine residue, which were found in this protein (Figure 4). VvLCC3 is indicated to be a laccase family member by four copper binding sites with conserved histidine and cysteine residues.

**Figure 5:**
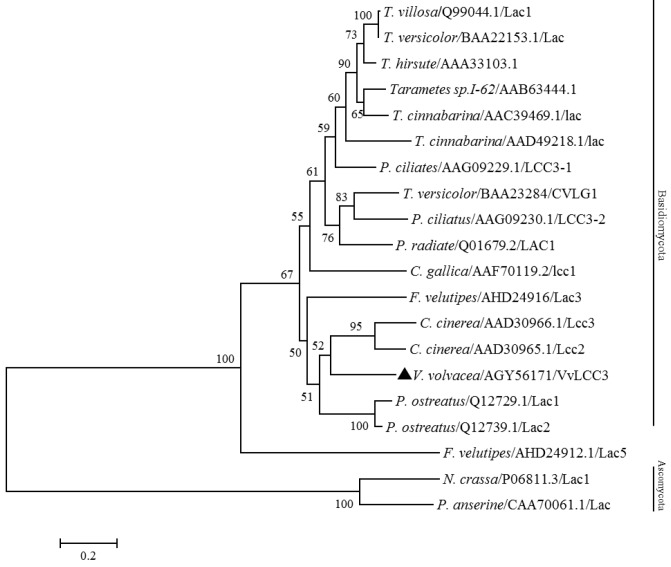
Phylogenetic tree of *V. volvacea* VvLCC3 protein sequences with amino acid sequences of laccases identified in other fungi. *P. ostreatus*: *Pleurotus ostreatus*; *T. versicolor*: *Trametes versicolor*; *T. villosa*: *Trametes villosa*; *T. hirsute*: *Trametes hirsute*; *T. cinnabarina*: *Trametes cinnabarina*; *P. ciliates*: *Polyporus ciliates*; *P. radiate*: *Phlebia radiate*; *N. crassa*: *Neurospora crassa*; *P. anserine*: *Podospora anserine*. The tree was constructed on http://www.phylogeny.fr/ and, bootstrap values (100 replications) higher than 50% are indicated for the nodes. The triangle indicates *V. volvacea* VvLCC3 protein. Nominated outgroup was Ascomycota (including *P. anserine* and *N. crassa*).

Phylogenetic analysis demonstrated that all of the laccases we collected from Basidiomycota or Ascomycota divisions formed independent clades, which is in agreement with previous studies [26]. The VvLCC3 clustered with laccase Lcc2 and Lcc3 from *C. cinerea*, as previously reported [15,16,25], and is slightly further from *P. ostreatus* Lac1 (Q12729.1) and Lac2 (Q12739.1) (Figure 5). This is a precision that was not revealed in Lettera (2010) phylogenic tree built with protein sequences where the laccases of the three species were in an equivalent position in a rake [16]. But bootstrap values here are too low to support these nodes in the phylogenic tree. Nevertheless this structure of the phylogenetic tree, indicating a high similarity between VvLcc3 and Cci-lcc3, conforms to the analysis of intron positions (Figure 3), and is congruent with the tree built by Ahlawat *et al.* (2011 (see Figure 2 and Figure 4)) with intron positions [25]. In addition, the BLASTP results revealed that the deduced protein product of *Vvlcc3* showed high identity with other fungal laccases, such as, *C. cinerea* (AAD30965.1, 68%), *L. bicolor* (XP_001874989, 63%), *Coprinus comatus* (AFD097049.1, 65%), *Stropharia aeruginosa* (AFE48786.2, 64%), *Cyathus bulleri* (ABW75771.2, 65%), *C. cinerea* (AAD30966.1, 65%), and *F. velutipes* (ADX07319.1, 63%). On the contrary, intron positions in laccase *Lcc1* and *Lcc2* [25], (corresponding to *vv-lac9* and *vv-lac5* in Bao nomenclature), and in all other *V. volvacea* laccase genes except *vv-lac6* and *vv-lac11* (=*Vvlcc3* in the present study) indicate a divergence of these laccase genes with the other basiomycete laccase genes, which is confirmed in the neighbor joining tree published by Lettera *et al.* (2010) [16].

### 2.4. Heterologous Expression of Vvlcc3 in P. pastoris

**Figure 6:**
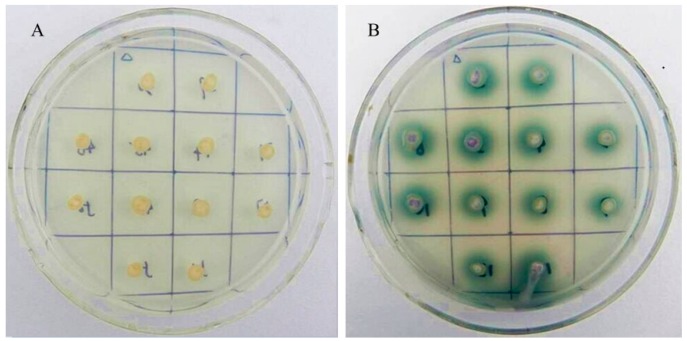
Screening of positive transformants with BMM medium. (**A**) *pPIC9K* acting as negative control; (**B**) *pPIC9K*-*Vvlcc3*.

The *Vvlcc3* cDNA, without the native signal sequence, was inserted downstream of the α-factor secretion signal of the *P. pastoris* expression vector pPIC9K. The *pPIC9K-Vvlcc3* and pPIC9K sequences were digested with Sac I. Then, the *pPIC9K-Vvlcc3* and pPIC9K were transformed into *P*. *pastoris* GS115 and screened by Buffered minimal glycerol (BMM) plates. The positive transformants containing *pPIC9K*-*Vvlcc3* produced green zones around their colonies, whereas those containing pPIC9K did not display any color changes (Figure 6). A green-surround colony was chosen for further experiment.

**Figure 7:**
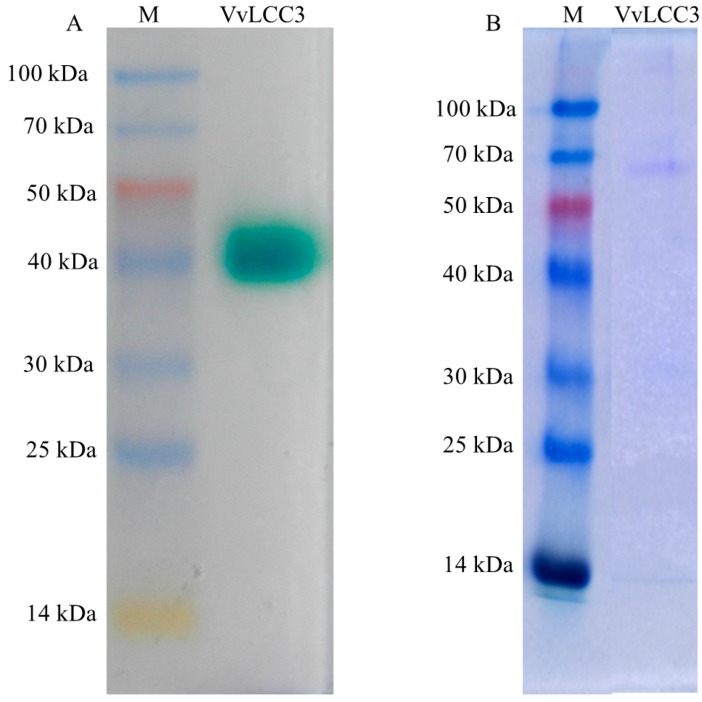
Native-PAGE and SDS-PAGE of purified VvLCC3 secreted by *P. pastoris*. M: protein marker, VvLCC3: laccase expressed in *P. pastoris* GS115. (**A**) Native-PAGE of purified VvLCC3 secreted by *P. pastoris*; (**B**) SDS-PAGE detection of purified VvLCC3. During the process of electrophoresis, the mobility of proteins depends not only on the size of proteins, but also on the charge of proteins in protein analysis using native PAGE. Since SDS-PAGE overestimates the molecular masses, the band sizes are different in the two types of gels.

The laccase activity peaked after 21 days of cultivation (296.83 U/L), and heterologous VvLCC3 was purified and detected by native-polyacrylamide gel electrophoresis (native-PAGE) and sodium dodecyl sulfate-polyacrylamide gel electrophoresis (SDS-PAGE), both of which displayed a single band (Figure 7). The molecular weight of purified VvLCC3 was approximately 65 kD, which is consistent with the features of the previously characterized fungal laccases (60 to 80 kDa) [27].

## 3. Discussion

Laccases were first discovered in the Japanese lacquer tree *Rhus vernicifera* (reviewed by Thurston) [27]. Subsequently, laccases have also been identified in other plants [28,29], in various fungi [30,31,32,33], in bacteria [34,35,36,37,38], and in insects [39]. Laccases carry out a diverse array of biological roles. In plants, laccases are involved in lignin biosynthesis [40]. In bacteria, laccases appear to participate in the protection of the spores against stress factors such as hydrogen peroxide or UV radiation [36].

In fungi, laccases are not only involved in the biosynthesis of pigments, lignin degradation, conidiation and sporulation but also related to lignin bioconversion, the development of fruiting bodies, and gill browning after fruit body harvest (reviewed by Kües *et al.*) [20]. In this study, the transcription of 11 laccase genes was analyzed during the process of stipe elongation in *V. volvacea*. Among the 11 genes, *Vvlcc3* exhibited the highest expression levels in every sample, and thus was cloned and expressed in *P. pastoris*. Analysis of the deduced protein showed that VvLCC3 carried four conserved sequence regions L1-L4 and showed high sequence identity with laccases from *C. cinerea*, *L. bicolor*, and *F. velutipes*, suggesting that the protein encoded by the *Vvlcc3* gene was a laccase [41]. The phylogenetic tree provided further evidence for that *Vvlcc3* was a *sensu stricto* laccase gene. In addition, the presence of green zones around colonies expressing the laccase protein on BMM plates proved that the *Vvlcc3* gene encoded a functional laccase.

The stipe of *V. volvacea* is very small in the button stage, and stretches in the egg stage, and then extends to almost full length in the elongation stage [2]. The expression level of the *Vvlcc3* gene, reached a peak level in the elongation stage, consistent with the change of stipe tissues during the development of the *V. volvacea* fruit body. Furthermore, the transcription pattern of *Vvlcc3* was similar to the trend in expression of *Fv-lac3* and *Fv-lac5* involved in stipe elongation in *F. velutipes*. This suggested that *Vvlcc3* could be involved in stipe elongation in *V. volvacea*, as the genes specified above could be in *F. velutipes* [8]. Phylogenetic analysis of VvLCC3 showed a clustering of sequences that could reflect their function, and it is possible to hypothesize that the clade containing *F. velutipes* Lcc3 and VvLCC3 contain genes involved in stipe elongation, but this hypothesis need to be verified.

Extracellular laccases are reported to be correlated with pigment production and lignin bioconversion [20]. Also, extracellular laccases can crosslink hyphal walls together during the initiation of primordium [42] and, may continue to act on the surfaces of mycelium during fruiting body development [43]. As a peptide signal was present, VvLCC3 could function at extracellular levels. Fv-Lac5 could play the same role as VvLCC3 as the two genes are expressed at the same stage and have a peptide signal, unlike Fv-Lac3.

Knock out experiments, which are useful for studying the function of genes, should be performed to further confirm the role of *Vvlcc3* in stipe elongation. However, it is still a challenge to knock out genes in *V. volvacea* [1]. Transcription pattern analysis of genes during the development of the fruiting body could provide the basis for elucidating the different parameters of the stipe elongation of the fruiting body in *V. volvacea*, given that stipe elongation is considered to be the cause of rupture of the universal veil and, in turn, the reduction of commodity value [1]. In summary, our preliminary results may be useful for the promotion of *V. volvacea* cultivation.

## 4. Experimental Section

### 4.1. Strains and Vectors

Strain H1521 (heterokaryon), stored at the Agricultural Culture Collection of China (accession no.ACCC52633), was provided by the Mycological Research Center of Fujian Agriculture and Forestry University.

*Escherichia coli* DH5α (TIANGEN, Beijing, China) was used as the host for the cloning procedures. PZeroBack/Blunt Vector (TIANGEN, Beijing, China) was used to subclone the cDNA fragment for sequencing. The *pPIC9K* plasmid with an alpha-factor signal peptide and *Pichia pastoris* GS115 (Mut^+^ His^−^) were purchased from Invitrogen (Carlsbad, CA, USA).

### 4.2. Isolation of Total RNA

*V. volvacea* strain H1521 was cultured on rice saw compost [21]. Samples at different development stages were harvested according to the method of Tao *et al*. [1] and frozen in liquid nitrogen.

Total RNA was isolated from samples using an E.Z.N.A.™ Plant RNA Kit (OMEGA, Stamford, CT, USA) according to the manufacturer’s instructions. The first strand of cDNA was synthesized using TransScript^®^ One-Step gDNA Removal and cDNA Synthesis SuperMix (Transgen, Beijing, China). All cDNA was stored at −20 °C for the subsequent experiments.

### 4.3. Transcription Pattern Analysis of Laccase Genes in V. volvacea with DGE Data

To construct and sequence the digital gene expression (DGE) libraries, the mRNA extracted from stipes of four developmental stages of the fruiting body was submitted to BGI (Shenzhen, China). And the method used for constructing and sequencing of the DGE tag libraries was described by Tao *et al*. [1]. Then, we deposited the DGE data in the NCBI’s GEO database (accession number: GSE43297) and, analyzed the expression levels of laccase genes in *V. volvacea* using the DGE data. Briefly, the expression of these genes was calculated based on the number of tags uniquely mapped to the cDNA of laccase genes, which were then normalized to Transcripts per Million clean tags (TPM) [23].

Further, qRT-PCR was performed to verify the expression of *Vvlcc3* analyzed using the DGE data. SYBR Premix Ex TaqTM II (Tli RNaseH Plus) (Takara, Tokyo, Japan) was used in this study. A total reaction volume of 25 µL was prepared according to the manufacturer’s protocols. The qRT-PCR programmer was as follows: initial denaturation 95 °C for 30 s, 40 cycles of 95 °C for 5 s and 60 °C for 30 s. The primers for *Vvlcc3* and glyceraldehyde-3-phosphate dehydrogenase (*gapdh*) genes [1,44], used as an internal standard, were designed with Primer Premier 5.0 (Table S3). And then, we used the 2^−ΔΔ*C*t^ method for qRT-PCR data analysis [45]. All experiments were conducted in triplicate.

### 4.4. Cloning of Vvlcc3 cDNA, Construction of Expression Vector and Transformation

To clone the *Vvlcc3* *cDNA*, PCR was performed with primers lcc3OF and lcc3OR, and the cDNA from the button stage as a template. The PCR temperature program was as following: initiation step was 95 °C for 3 min; next step was 35 cycles DNA amplification, and each cycle contained 95 °C for 30 s, 58 °C for 30 s and 72 °C for 4 min; and a final extension step at 72 °C for 10 min. The reaction mixture for PCR was as follows: 2 μL of cDNA, 2.5 μL of dNTP Mixture, 2.5 μL of Pfu buffer (with MgSO_4_), 0.5 μL of Pfu DNA Polymerase, 1 μL each of forward and reverse primers, and 15.5 μL of ddH_2_O. The PCR products were then cloned into pZeroBack/Blunt Vector (Tiangen, Beijing, China) for sequencing (Sangon Biotech, Shanghai, China).

The open reading frame (ORF) of *Vvlcc3* without the native signal peptide sequence was flanked by Avr*II* and Not*I* restriction sites at the 5’- and 3’-ends respectively, with PCR using lcc3-F-Avr*II* and lcc3-R-Not*I* primers. We subcloned the fragments into a pZeroBack/Blunt Vector and then digestion was performed with restriction enzymes Avr*II* and Not*I*. Finally, the fragments were ligated into the corresponding sites of pPIC9K (*Pichia pastoris* expression vector) and we named the validated recombinant plasmid as *pPIC9K-Vvlcc3*.

Both the recombinant plasmids *pPIC9K-Vvlcc3* and *pPIC9k* without *Vvlcc3*, which were used to prepare negative control strains, were linearized using the restriction enzyme Sac*I* and transformed into *P. pastoris* GS115 by electroporation (Invitrogen). The transformants were selected on Minimal Dextrose medium (MD) agar plates (1.34% yeast nitrogen base (YNB), 4 × 10^−5^ biotin, 2% dextrose) at 28 °C, after which His^+^ transformants were screened using direct PCR with the lcc3-F-Avr*II* and lcc3-R-Not*I* primers.

### 4.5. Expression, Purification and Analysis of Heterologous VvLCC3

The His^+^ transformants were transferred to Buffered Glycerol-complex Medium (BMGY) agar plates (2% peptone, 1% yeast extract, 1.34% YNB, 1% glycerinum 4 × 10^−5^ biotin, and 1% glycerinum) at 28 °C for 2 days. BMM agar plates (1.34% YNB, 4 × 10^−5^ biotin, 0.5% (*v*/*v*) methanol, 0.1 mM CuSO_4_ and 0.2 mM ABTS (2,2′-Azino-bis(3-ethylbenzothiazoline-6-sulfonic acid)), 100 mM of potassium phosphate (pH 6.0)) [46] were applied to screen the transformants that were secreting *VvLCC3* according to the presence of a green zone around the His^+^ transformant colonies.

We inoculated the *Vvlcc3* transformants in 100 mL BMG (prepared according to the instruction by Invitrogen) at 28 °C and 150 rpm until the OD_600_ value reached 10. Then, the *P. pastoris* cells were collected by centrifugation at 1500× *g* for 5 min, and resuspended with 50 mL BMM (containing 0.3 mM CuSO_4_ and 0.8% alanine) at 28 °C and 150 rpm [46]. Methanol was added daily to a final concentration of 0.5% (*v*/*v*) to induce expression of *Vvlcc3* and 1 mL of culture was taken daily from the flask. Supernatants were collected by centrifugation prior to the measurement of laccase activity according to a previously described method [47]. We defined one unit of laccase activity as the enzyme catalyzed the oxidation of 1 μmol 2,2’-Azino-bis(3-ethylbenzothiazoline-6-sulfonic acid) (ABTS) min^−1^ [48]. To identify laccase, native-PAGE was implemented with 8 mL of separating gel (12% (*w*/*v*)) and 2 mL of stacking gels (5% (*w*/*v*)). After electrophoresis, we stained the protein band with laccase activity with 1 mM ABTS in 0.1 M acetate buffer (pH 5) [49].

After performing of the experiments mentioned above, supernatants with the highest laccase activity were harvested by centrifugation at 10,000× *g* for 10 min and concentrated to a volume of 5 mL using PEG4000. The concentrate solution was applied to a Sephadex G-15 column (10 mm × 300 mm) pre-equilibrated with 0.05 M phosphate buffer (pH 6.8). It was eluted using the same buffer and the eluted protein was detected by 1 mM ABTS in 0.1 M acetate buffer (pH 5), after which the eluted proteins were applied to a DEAE-cellulose column (10 mm × 300 mm, DE52) pre-equilibrated with 0.05 M phosphate buffer (pH 6.8). After wash with 500 mL of the same buffer, the unbound proteins were removed from the column. Subsequently, the bound laccase was eluted with sodium chloride solution in gradient concentration (from 0.05 to 0.45 M). Eluted proteins were pooled, concentrated to 2 mL using PEG4000, and stored at −20 °C for further investigation.

SDS-PAGE (10% *w*/*v*) was conducted to investigate the purified protein. After which, we stained the protein band with Coomassie Brilliant Blue R-250 at room temperature for 2 h. Protein molecular weight markers (Takara) were used to estimate the molecular weight of heterologous VvLCC3.

### 4.6. Protein Sequences Analysis and Phylogenetic Tree Construction

The basic physical and chemical characteristics of VvLCC3 were analyzed using ExPASy Protpara (http://www.expasy.ch/tools/protparam.html) [49], whereas the signal peptide was predicted by signalP 4.1 Server (http://www.cbs.dtu.dk/services/SignalP) [50]. The analysis of the amino acid conservation domains was performed using InterProScan (http://www.ebi.ac.uk/Tools/pfa/iprscan/) [51]. NetNGlyc 1.0 Server (http://www.cbs.dtu.dk/services/NetNGlyc/) was applied to the analysis of the *N*-glycosylation sites [52].

To generate the phylogenetic tree, the amino acid sequences of the 19 typical laccases from other fungi were obtained from NCBI according to the approach of Valderrama *et al*. [53]. Then, these sequences and the predicted laccase amino acid sequences of laccase encoded by *vvlcc3* gene were aligned with ClustalX 1.83 [54]. A maximum likelihood method performed on Phylogeny.fr (www.Phylogeny.fr) [55,56,57,58] was applied to construct a phylogenetic tree and bootstrap analysis was implemented with 100 replicates.

## Supplementary Materials

Supplementary materials can be found at http://www.mdpi.com/1422-0067/16/12/26111/s1.
