# Supplementary material for: Cloning and Expression Analysis of Vvlcc3, a Novel and Functional Laccase Gene Possibly Involved in Stipe Elongation
Source: Int J Mol Sci. 2015 Dec 1;16(12):28498–509. doi: 10.3390/ijms161226111 (PMC4691058; doi:10.3390/ijms161226111)
Supplement: Supplementary file 1 [file ijms-16-26111-s001.pdf]

Supplementary Materials: Cloning and Expression Analysis of Vvlcc3, a Novel and Functional Laccase Gene Possibly Involved in Stipe Elongation

Yuanping Lu, Guangmei Wu, Lingdan Lian, Lixian Guo, Wei Wang, Zhiyun Yang, Juan Miao, Binzhi Chen and Baogui Xie

Table S1. GenBank accession number of laccase genes of *V. volvacea* strain PYd21.

PYd21	V23	Identity	*E*-Value	PYd21	V23	Identity	*E*-Value
Vvlcc1 (KF365489)	*vv-lac9*	98%	0	*Vvlcc7* (KF365495)	*vv-lac2*	99%	0
Vvlcc2 (KF365490)	*vv-lac5*	98%	0	*Vvlcc8* (KF365496)	*vv-lac4*	99%	0
Vvlcc3 (KF365491)	*vv-lac11*	100%	0	*Vvlcc9* (KF365497)	*vv-lac6*	99%	0
Vvlcc4 (KF365492)	*vv-lac3*	99%	0	*Vvlcc10* (KF365498)	*vv-lac7*	99%	0
Vvlcc5 (KF365493)	*vv-lac1*	99%	0	*Vvlcc11* (KF365499)	*vv-lac10*	99%	0
Vvlcc6 (KF365494)	*vv-lac8*	99%	0				

Table S2. The number of tags mapped on laccase genes at different developmental stages (from button stage to maturation stage).

	Vvlcc1	*Vvlcc2*	*Vvlcc3*	*Vvlcc4*	*Vvlcc5*	*Vvlcc6*	*Vvlcc7*	*Vvlcc8*	*Vvlcc9*	*Vvlcc10*	*Vvlcc11*	**Total Clean Tag Number**
BU	0	11	49	28	5	0	0	0	1	0	5	5,612,361
EG	2	2	425	87	8	3	0	0	9	0	8	5,978,522
EL	0	0	551	59	0	4	0	0	7	0	3	5,972,497
MA	0	7	162	67	0	6	0	0	3	0	0	5,967,968

Table S3. Primers used in this study.

Primer	Nucleotide Sequence
P*gpdh*F	5′-ATTGGCGTGGTGGTCGTAG-3′
P*gpdh*R	5′-ACGGAAACATCAAGGGTAGGG-3′
P*lcc3*F	5′-TATTCACTGGCACGGATTCTTC-3′
P*lcc3*F	5′-TCATCAGGATCATAAACGACGAG-3′
lcc3OF	5′-ATGCTGTCGCCGACCTTGGTC-3′
lcc3OR	5′-TTATTGGTCGAAAGTCTCGAC-3′
lcc3-F-Avr*II*	5′-CCTAGGATAGGGCCGGTCACAGAGCT-3′
lcc3-R-Not*I*	5′-GCGGCCGCTTGGTCGAAAGTCTCGACAGGG-3′
